# A feasibility trial of olanzapine for young people with Anorexia Nervosa (OPEN): clinicians’ perspectives

**DOI:** 10.1186/s40337-024-01106-9

**Published:** 2024-09-27

**Authors:** Vanessa Kellermann, Ece Sengun Filiz, Olena Said, Jessica Bentley, Joel W.T. Khor, Mima Simic, Dasha Nicholls, Janet Treasure, Ulrike Schmidt, Hubertus Himmerich, Vanessa Lawrence

**Affiliations:** 1 Department of Health Service and Population Research, Institute of Psychiatry, Psychology & Neuroscience, King’s College London, London, UK; 2 Division of Psychiatry, Department of Brain Sciences, Imperial College London, London, UK; 3 Centre for Research in Eating and Weight Disorders (CREW), Department of Psychological Medicine, Institute of Psychiatry, Psychology & Neuroscience, King’s College London, London, UK; 4 Department of Psychological Medicine, Institute of Psychiatry, Psychology & Neuroscience, King’s College London, London, UK; 5 St George’s Eating Disorders Service, South West London and St George’s Mental Health NHS Trust, London, UK; 6 South London and Maudsley NHS Foundation Trust, London, UK

**Keywords:** Olanzapine, Anorexia nervosa, Feasibility study, Qualitative, Survey, Lived experience

## Abstract

**Background:**

The OPEN feasibility trial testing olanzapine in anorexia nervosa (AN) in young people (YP) was not successful due to poor recruitment. This study aims to understand clinicians’ views and experiences of using olanzapine in AN and the challenges in implementing the trial in National Health Service (NHS) clinical settings.

**Methods:**

We conducted qualitative interviews with eating disorders (ED) clinicians involved with the study (*n* = 11). Framework analysis was applied to qualitative data to identify barriers and facilitators to recruitment and study implementation. A web-based semi-structured Qualtrics survey was administered to ED clinicians (*n* = 24). Findings from the survey were used to corroborate and expand on the information derived from qualitative interviews.

**Results:**

Qualitative analysis identified four main themes: (1) *Acknowledging Service User (SU) / Family Concerns*, (2) *Prioritising person-centred care*, (3) *Limited Service Capacity* and (4) *Study eligibility criteria*. Subthemes are outlined accordingly. Clinicians appeared confident addressing SU concerns around olanzapine in clinical discussions, but timing was critical, and olanzapine was considered one aspect of treatment that needed to align with their holistic approach. Service pressures restricted opportunities for recruitment and the ability to offer regular review. At the same time, some YP were ineligible for the trial, as they were already taking olanzapine, or needed to be prescribed it more promptly than the study procedures allowed. Survey findings underlined confidence in prescribing and informing on olanzapine, the various possible benefits of olanzapine besides weight gain, and the importance of therapeutic alliances and informed consent. Both data sets highlight the need for further evidence on long-term safety, side effects and efficacy of olanzapine use for AN. Where clinical service capacity is at a premium, research implementation is not prioritised, particularly in intensive clinical settings.

**Conclusions:**

Findings provide first-hand insight into individual and systemic challenges with research implementation in the NHS, which need to be considered when designing future clinical research studies. We emphasise a person-centred approach when discussing olanzapine to consider a holistic recovery from AN beyond weight-gain as an isolated outcome for improvement.

**Supplementary Information:**

The online version contains supplementary material available at 10.1186/s40337-024-01106-9.

## Abstract (plain language summary)

Although olanzapine, an atypical antipsychotic medication, is commonly used in eating disorder services across the world, it is not currently recommended by clinical guidelines in the United Kingdom. We interviewed clinicians working in eating disorder services that took part in a research study looking at olanzapine for young people with anorexia nervosa (AN), and published a survey asking clinicians about their experiences with and views on using olanzapine for AN. The lack of official long-term evidence concerned both clinicians and services users (SU)s. Many clinicians suggested that people with AN might be fearful of weight-gain as a side effect from olanzapine and therefore declined to take part in the study. Having a good relationship built on trust and consistency was deemed very important by clinicians to talk about medication and to provide good health care that focuses on the needs of SUs. Clinicians explained that it was a challenge to introduce the study to SUs in their day-to-day work, as mental health services are increasingly overburdened. Clinicians were also clear that SUs should only be prescribed olanzapine within the study if it was at the right time for them and fitted their treatment pathway.

**Supplementary Information:**

The online version contains supplementary material available at 10.1186/s40337-024-01106-9.

## Background

Anorexia nervosa (AN) is a mental disorder associated with significant morbidity and mortality. With two thirds of onsets occurring between 14- and 25-years old [1], YP are a particular group of interest for intervention, with highest chances of remission if treated promptly [2].

Olanzapine is an antipsychotic recommended by various guidelines globally [3] for short-term treatment of obsessional thinking in AN but not for weight restoration due to insufficient evidence base for its efficacy and safety. Four randomised controlled trials (RCTs) to date show some significant effect on weight gain in people with AN [2, 4–6] with less clarity in adolescents [7, 8], on safety and acceptability, and inconsistent effects on psychopathology. Consequently, the most recent World Federation of Societies of Biological Psychiatry (WFSBP) guidelines update in 2023 on the pharmacological treatment of eating disorders (ED) gave only a limited recommendation for adjunct olanzapine to achieve weight gain [9]. Nevertheless, antipsychotics are commonly used off-label for AN [10, 11]. It remains unclear how clinicians make decisions about its use, how they perceive its safety and efficacy, and address uncertainty in discussions with patients and carers.

Olanzapine for young PEople with aNorexia nervosa (OPEN) was designed as an open-label feasibility study targeting patients with AN, aged 12–24 years, treated with olanzapine and followed up for 12 months. ‘Young people (YP)’ will refer to said age-group throughout this article. The study was registered with the International Standard Randomised Controlled Trial Number (ISRCTN80075010) [12]. Primary aim of OPEN was to assess feasibility of a future definitive trial on olanzapine in YP with AN, with qualitative feasibility parameters testing the acceptability of intervention and study design by participants, their family/carers and clinicians [12].

From June 2022 to May 2023, only 20 of 55 intended participants were recruited to OPEN across ten study sites, and only 13 completed a follow up assessment at 6 or 12 months [13]. Here, we examine the reasons underlying poor recruitment and retention from the perspective of clinicians in eating disorder services.

Research activity across United Kingdom (UK)’s National Health Service (NHS) has decreased in recent years, indicating prioritisation of clinical needs over research. Previous ED trials showed impact of systemic-level barriers on trial recruitment, particularly in overstretched services [14]. Exploring those barriers to research implementation is necessary to inform the direction of future research on olanzapine in AN.

## Methods

### Study design

Our aim was to investigate the perceived acceptability and feasibility of olanzapine’s use in AN and design of OPEN via 1. individual interviews with clinicians working at OPEN study sites 2. an online survey of clinicians across ED services in the UK. We adopted an exploratory mixed methods sequential design [15], drawing from emerging themes in initial interviews and feedback from recruiting sites, to design an online survey capturing a wider understanding.

### Qualitative interviews

#### Recruitment and participants

Clinicians from participating sites were invited via e-mail to an individual interview about their views and experience of olanzapine for YP with AN within the context of OPEN. Purposive sampling aimed for a range of professional backgrounds with some exposure to and/or experience with olanzapine and proximity to OPEN. Socio-demographics were collected for self-reported gender, ethnicity, professional role and years of work in ED services.

#### Data Collection

Staff were interviewed online via Microsoft Teams by a researcher with lived experience of atypical AN. Staff were given a Participant Information Sheet and provided written consent via email. A semi-structured topic guide was developed by the research team and explored: staff experiences of working in an ED service, including treatment priorities and concerns; experiences, hopes and reservations around use of olanzapine for YP with AN; and potential challenges and enablers to implement OPEN. Researcher used the topic guide flexibly to follow participants’ concerns and kept an analytical diary to capture initial reflections on issues raised, to support reflexivity and note questions and hunches to explore in subsequent interviews.

#### Data analysis & trustworthiness

Interviews were transcribed verbatim, anonymised, and analysed using framework analysis [16]. VK first familiarised herself with the data, reading each transcript repeatedly before developing an initial thematic framework based on barriers and facilitators to recruitment, reflecting both the topic guide (e.g. concerns around olanzapine) and emergent themes (e.g. monitoring treatment safety). Using NVivo 14 [17] topics were coded, data reviewed for coherence, and thematic framework revised before key themes, sub-themes and data extracts were summarized in charts to facilitate subsequent interpretation.

Regular discussions between VK and VL and wider study team, mind maps, and conference presentations and feedback, helped to develop and revise more analytical themes (e.g. taking a holistic approach) and evaluate their fit. Bringing a personal perspective provided VK with sensitivity and awareness to past and current difficulties within clinical space regarding medication use in EDs when collecting and analysing data. Other authors also drew on their respective backgrounds (e.g. ESF, Academic Clinical Fellow in Child and Adolescent Psychiatry; VL, Social Scientist) to consider alternative interpretations, which were constantly checked against the data.

### Online survey

Preliminary themes from clinician interviews, and feedback from study team and clinicians at sites, were used to create an online survey gathering views from various clinicians in ED services across the UK, about use of olanzapine for AN. This allowed examination of the strength and wider applicability of our analysis on barriers and facilitators of studying olanzapine for AN.

The survey was advertised on the British ED Society’s noticeboard, the Royal College of Psychiatrists’ ED Conference (November 2023), ED faculty’s social media account and in OPEN study newsletter for sites. All participants were given information and consented before providing their anonymous answers to questions. It was designed on Qualtrics, covering a broad range of questions in different styles, i.e., multiple choice questions, scaling questions, and free text forms.

The survey collected information on the characteristics of responders, e.g. gender, professional role/grade, professional setting and age group, involvement with OPEN/other research, and lived experience of ED. Survey questions examined their clinical practice around Olanzapine for AN, their views on the impact of therapeutic alliance on discussions around medication, appropriateness and evidence base of Olanzapine and how this affects their decision-making and Olanzapine`s acceptability to YP. Perspectives regarding clinical research for Olanzapine in AN, and its relation to service capacity, and general study procedures and how they differed from routine clinical practice were investigated.

Responders could skip questions and answered different follow-up questions based on their background (e.g., scope of professional activities or involvement in OPEN).

## Results

### Participant characteristics

#### Qualitative interviews

Eleven clinicians were interviewed, with recruitment continuing until the sample provided sufficient depth and breadth of perspectives. Nine interviewees identified as female and ten as white. Nine had a medical background with majority working in outpatient settings (Table 1).

**Table 1:** Staff demographics table

Staff ID	Gender	Ethnicity	Professional role	Environment	Years in ED work
S1	Female	White Other	Speciality Doctor	Outpatients	2.5
S2	Female	White Other	Consultant Psychiatrist	Outpatients, Inpatients, Day Care	25
S3	Female	Asian	Consultant Psychiatrist	Day Care	5
S4	Female	White British or Irish	Speciality Doctor; Registrar	Outpatients	1.1
S5*	N/A	N/A	N/A	N/A	N/A
S6	Female	White British or Irish	Assistant Psychologist	Outpatients	1.4
S7	Female	White Other	Trainee Psychiatrist	Outpatients, Inpatients, Day Care	0.5
S8	Female	White British or Irish	Senior Counselling Psychologist	Outpatients	3
S9	Female	White British or Irish	Speciality Doctor	Outpatients	0.6
S10	Male	White British or Irish	Lead Clinician; Registered Mental Health Nurse	Outpatients	37
S11	Female	White British or Irish	Senior ED Specialist; Nurse (Prescriber)	Outpatients	1.5
S12	Male	White Other	Consultant Psychiatrist	Outpatients	2

*Participant withdrew from study post recruitment

#### Survey

Twenty-six clinicians agreed to take part in survey (Seet Table 2). Twenty-three identified as female, two as male, one preferred not to report gender. Nine were psychiatrists working in ED services (four adult and four child and adolescent psychiatrists respectively, one higher specialist psychiatry trainee), four psychologists, seven nurses, six allied health professionals, such as dieticians. Four reported involvements with OPEN and were psychiatrists. Only one participant reported involvement with other research with olanzapine. Twenty-four worked in ED services: 17 in outpatient, 15 in inpatient, three in daycare services; nine in a combination of outpatient and inpatient settings; three across all settings. Fifteen worked with adults, 18 worked with children and adolescents, and seven worked across all ages. Nine reported lived experience of ED: five had lived experience as a patient/SU, two as a carer, and two both as a carer and a SU.

**Table 2:**
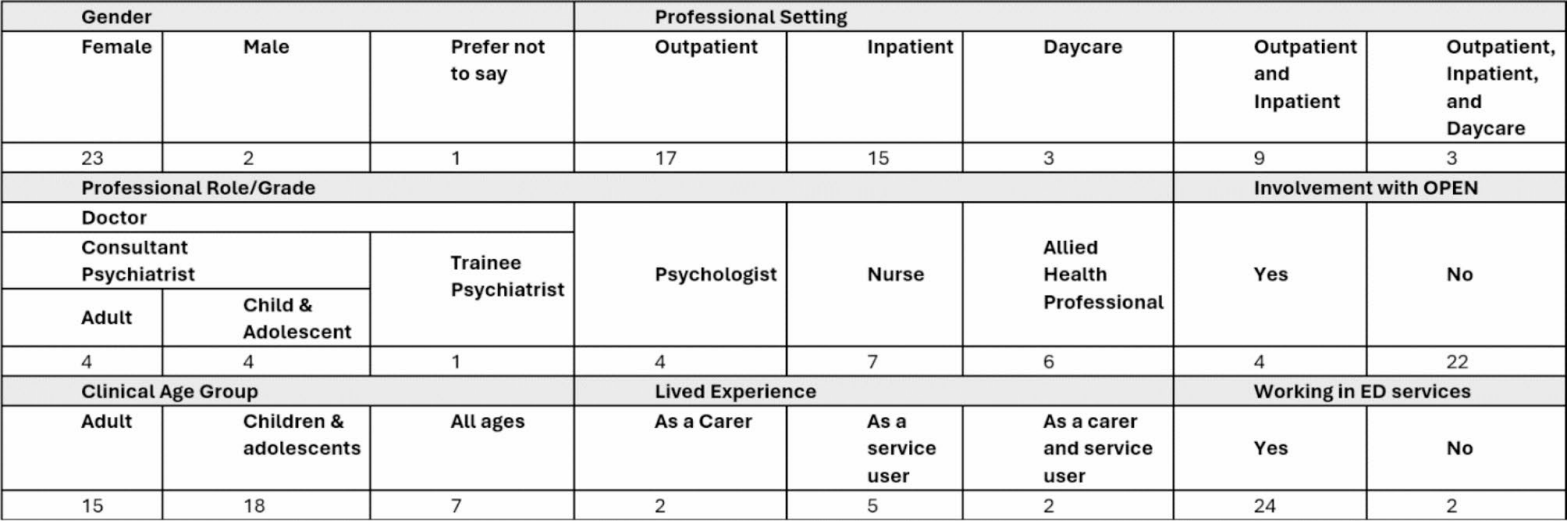
Survey participant characteristics

Clinicians commonly identified OPEN, and a potential subsequent RCT investigating efficacy and safety of olanzapine in YP with AN, as important and well-needed. All responders in the survey expressed a clinical trial of olanzapine as adjunct treatment for AN as “necessary”. Yet our framework analysis identified multiple challenges to this research, captured in themes presented below. Survey results are presented underneath relevant themes examining their wider applicability.

### Theme 1: Acknowledging service user / family concerns

Concerns of SUs and families around olanzapine were identified as a potential barrier to recruitment, needing acknowledgement in clinical conversations.

#### Evaluating available evidence

Clinicians noted many SUs and/or families conducting their own research before deciding, drawing on current research, clinical guidelines, and sources outside the academic and medical sphere, including internet forums and narrative/personal accounts. Over half felt that this provoked concern around side effects, lack of long-term evidence in YP and/or uncertainty regarding *how* olanzapine works.

*“Obviously they look things [up] online*,* […] and that is I guess one of the issues as well is that there’s not actually much evidence up there for AN*,*”* (S1, Speciality Doctor).

In the survey, over half of clinicians (9/15) agreed that available evidence for efficacy impacted the acceptability of olanzapine by SUs and/or carers. The remaining clinicians were neutral.

#### Fear of weight gain

Independent research by SUs was suggested to exacerbate anxiety about increased appetite and weight gain. Most interviewees (8/11) identified this as a major barrier to recruitment. Clinicians contextualised this by explaining that many YP with AN presenting to services with an ambivalence and/or resistance towards recovery. Some contrasted patient perspectives on antidepressants to improve mood with antipsychotics/olanzapine, which was seen as more threatening as thought to ‘directly’ treat AN:

*“Everyone wants to feel better so the anti-depressant’s less of a big jump*,* but not everyone wants to get rid of their anorexia.”* (S8, Senior Counselling Psychologist).

Notably, no other side effects were mentioned as important to SUs:

*“They don’t mind the sedation. They don’t mind the prolactin or any of the other side effects. It’s the weight gain that everyone gets very concerned about.”* (S12, Consultant Psychiatrist).

Weight gain was specifically mentioned as “the biggest side effect” and a potential “barrier to concordance” by a clinician in the survey. Patients that were pre-occupied with potential weight gain, and/or uneasy with increased appetite, were considered less likely candidates for olanzapine.

#### Stigma surrounding antipsychotics

Clinicians (7/11) explained that families and SUs often held broad concerns around psychotropics, and specifically around antipsychotics. Social stigma was seen to create hesitancy among SUs and families. Most interviewees considered SUs and loved ones as more fearful of anti-psychotics than anti-depressants:

*“They might not want their friends to know that they’re taking an anti-psychotic; it sounds quite dramatic […]. People are a bit more comfortable with […] an anti-depressant than an anti-psychotic.”* (S8, Senior Counselling Psychologist).

Others saw illness itself as impacting on attitudes towards psychotropics generally; stating that *“A lot of people with anorexia have really strong values about feeling that they ought to sort things out for themselves and so taking a pill feels like giving in.”* (S10, Registered Mental Health Nurse).

#### Desperation outweighing concerns

Being in a precarious situation of acute need for *“anything that will help”* (S4) could be decisive for SUs and families. Though parents were sometimes described as initially reluctant, they were perceived to be less concerned about medication and potential side effects than their child, due to their desperation for recovery. One clinician identified caregivers of under 16s as notably keen to try a novel aspect of care and younger children more likely to be passive in decision-making, *“because things got to a point that they’re having to stop PE lessons*,* they’re going to A&E*,* their mum and dad are extremely worried…I don’t feel like they think they can say no.”* (S7, Trainee Psychiatrist).

*“The parents are often on board completely […]. They don’t have the same reservations.”* (S4, Speciality Doctor).

#### Providing reassurance and clear information

Almost all clinicians stressed the importance of presenting olanzapine and the study sensitively, providing SUs and families with reassurance about its usage and side effects. Highlighting intended dosage and duration, and explaining how it differs to treatment of psychosis, was deemed important. For some, this included transparent conversations with SUs about possible impact of olanzapine on appetite and weight gain, and participants’ ability to withdraw from the study. Trust was considered essential for these conversations. Two interviewees specifically explained how important it was not to “sell” olanzapine but to focus on understanding the individual needs and goals of each potential participant.

*“Often we’ll be thinking about using more motivational techniques*,* thinking about what their goals are*,* what they want to achieve […] then explaining how olanzapine can help to achieve those goals and being quite transparent and explaining that it’s not something that they need forever and that the weight gain*,* the appetite is not going to affect them long-term*,* is that this is an adjunct to help kick start their recovery.”* (S3, Consultant Psychiatrist).

*“I think the information here goes along with the support*,* […] this is not going to be ‘we dictate what’s going to happen to them’*,* […] we’re going to work with them to see how this affects them*,* and this is not written in stone*,* so we’re just doing this to help them recover from a really difficult point.”* (S7, Trainee Psychiatrist).

Some clarified that after participants agreed to olanzapine, there seemed little reluctance to participate in the study, as most SUs deemed it important to support the advance of evidence and potentially help others.

Among clinicians responding to the survey, most (12/16) felt comfortable explaining the rationale for starting olanzapine to patients and families. Fewer clinicians (8/18) expressed confidence about explaining its mechanism of action whilst another eight expressed lack of confidence and two remained neutral.

#### Including family & loved ones

Clinicians noted that the relationship between family and SUs could affect decision making.

*“Dad was very keen*,* which then made the young person not so keen. So*,* there’s that as well that certain people in the family may find this acceptable*,* but then it sort of doesn’t quite happen.”* (S2, Consultant Psychiatrist).

Eight out of 11 participants underlined the importance of including loved ones in these conversations and treatment process, especially due to SUs’ young age. Key people in their lives (parents, siblings, friends, etc.) were seen as integral to recovery and active participants in treatment.

*“We make it very clear the olanzapine is just a tool. It’s not the treatment. Family therapy is you supporting your child*,* using your strengths as a family. The olanzapine is not going to change any of that. It’s just going to make it a bit less difficult.”* (S1, Speciality Doctor).

### Theme 2: Prioritising person-centred care

#### Taking a holistic approach

Understanding the person as a whole, and prioritising their needs regarding treatment, was identified as an essential aspect of care by almost all clinicians. Some felt that suggesting olanzapine and study participation risked a negative impact on patient’s treatment and recovery. Multi-facetted needs, and a desire to protect the therapeutic relationship prevented some clinicians from mentioning OPEN. Where olanzapine had been successfully offered, it was considered an aspect of treatment that aligned with their holistic approach.

*“Where someone fits the bill*,* I will always talk to them about the study. But because it’s a treatment study it has to make sense in terms of where the person is at in their treatment.”* (S2, Consultant Psychiatrist).

Generally, clinicians underlined their intention to prescribe olanzapine with a focus on how it can benefit the individual on multiple levels (i.e., anxiety, sleep, or concentration), not only regarding anticipated weight-gain:

*“I see it very much as an adjunct […] That it could make people sleep a bit better or make them feel less bothered by some of their sort of anorexic cognitions […] So*,* for patients who are very revved up around anything to do with eating and so on*,* it can take the edge off that.”* (S2, Consultant Psychiatrist).

In the survey, multiple clinicians highlighted olanzapine’s usefulness in high severity or intractable AN, rigid and/or intrusive cognitions, anxiety, agitation around food/mealtimes, distress and sleep; e.g., *“If there is distress or overwhelming cognitions*,* it’s great - also good for restoration of sleep pattern”; “[It] has worked well in reducing anxiety symptoms in patients I work with”; “[I] have seen positive effects in reducing the rigidity of thinking and the degree of distress experienced”*,* “[It] has helped patients feel calmer and less agitated around meals and reduced intrusive thoughts”.*

Clinicians most frequent reasons for prescribing olanzapine were: “ED cognitions” (12/17), followed by “anxiety” and “lack of progress with other treatments” (10/17), sleep disturbances (5/17), “unsuitability of psychological interventions” and “patient preference” (4/17), “weight below a threshold”, “parent/carer initiative” and “distress/agitation” (2/17), and “medical complications” (1/17).

The majority of doctors (7/8) felt comfortable about starting a patient with AN on olanzapine and most non-medical clinicians (8/10) were comfortable about managing a patient with AN on olanzapine. Thirteen out of 17 reported feeling supported about managing the risks and side effects of olanzapine for AN.

When asked what made a patient a less likely candidate for olanzapine, clinicians listed the following: very young age, inability to or lack of consent of the patient or parent, binging-purging, cardiovascular instability and pre-existing cardiac conditions, deranged liver function tests, being prone to metabolic conditions that could be exacerbated by olanzapine, being normal in weight, reasonable response to therapeutic intervention or other medication, and fear of weight gain.

#### Prioritising immediate treatment needs

Clinicians explained that olanzapine and/or participation in OPEN needed to fit SU’s treatment trajectory, and other aspects of care sometimes took priority. Clinicians highlighted the importance of how and when to present olanzapine and the study without exacerbating anxiety and ensuring that SU was psychologically prepared to process weight gain and/ or well enough to engage with information on medication and study.

*“If there is a lot going on at the beginning when you first see someone and they’re crazy anxious and there’s a lot of uncertainty whether this person’s coming to the wards or outpatients or […] are they deteriorating? And you have a lot of stuff going on*,* then it’s probably quite hard to sensibly talk about this study.”* (S2, Consultant Psychiatrist).

#### Protecting the therapeutic alliance

Some underlined the importance of preventing unexpected disruption or change that could potentially damage trust and therapeutic alliance, and in turn, disrupt treatment as usual and/ or recovery progress. Concurrently, therapeutic rapport, and a trusting relationship in which SUs felt heard, was considered imperative for successfully introducing the study.

*“But the YP […] I was seeing regularly I often did see get better and I don’t think it’s because of anything in particular other than that they were trusting me because they got to know me*,* and they felt listened to. And I think that therapeutic alliance is like really powerful. And I definitely saw that.”* (S9, Speciality Doctor).

*“I think it also has an impact on how they perceive the helping system around them as well*,* because for many people with anorexia[…] feel suspicious of the people around them and worried about the impact. And so*,* if you’re completely upfront and give people*,* empower people to make choices for themselves*,* that can really help with engagement.”* (S10, Registered Mental Health Nurse).

The survey asked about clinicians’ level of agreement with an interviewee on *“We built quite a good rapport with the families*,* and they were […] both equally on board. […] They were open to having conversations about medication.”* All 11 clinicians outside OPEN and two out of three involved with OPEN agreed, suggesting that quality of rapport between a clinician and their patient/family was critical for their openness in discussing medication.

#### Clinical concerns around prescribing olanzapine in YP

A small group of clinicians (4/11) voiced concerns about prescribing olanzapine to very YP due to their ongoing physical development and the lack of long-term evidence regarding olanzapine’s use within this population. It was suggested that medication was generally prescribed with great caution to this group with clinicians prioritising other aspects of care, especially psychosocial support, and utilising medication in rare cases only.

*“[…] Our psychiatrists here*,* […] most psychiatrists are relatively conservative with a small C about prescribing to YP. […] [they] view medication as having a role*,* but not being of primary importance in helping people with eating disorders.”* (S10, Registered Mental Health Nurse).

In the survey, most clinicians (17/21) considered olanzapine appropriate as an adjunct treatment for AN. Most prescribers felt comfortable about starting (7/8) or managing a patient with AN on olanzapine (8/10).

When asked about whether they found the available evidence base for the efficacy of olanzapine for AN adequate, clinicians were mostly neutral (7/15) whilst 4 agreed and 4 disagreed. Most clinicians (9/15 and 11/15, respectively) agreed that current evidence base around the safety/side effects of olanzapine used for AN influenced their decision-making process and that evidence impacted on olanzapine`s acceptability to SUs and/or carers.

Multiple participants voiced dissatisfaction with limited evidence, both for weight restoration and improvement in psychopathology: *“I would like to see future research include impact of olanzapine on symptoms as well as weight”* and expressed their wish to see more clinical evidence on its safety and efficacy. One clinician reported uncertainty for cardiometabolic side effects making olanzapine inappropriate for AN, whilst others commented on its particular usefulness: *“[It] can be incredibly useful if the patient agrees to take it or it can be put down a tube”; “Excellent response with intractable conditions”; “Taking olanzapine has helped patients tolerate treatment who previously were too distressed to be able to tolerate the demands of treatment. It has felt almost miraculous at times to see the difference”.*

#### Stretched medical care

Some services were unable to offer regular medical reviews to every SU when needed, thus some SUs might not be seen by a psychiatrist frequently enough to monitor this treatment safely; and some may not be seen at all. Perceived differences existed between London and other areas that may have even lower capacity to provide the desired medical care.

*“We just had a really long list of people waiting for therapy and we don’t see them unless we are seeing them for physical health or they go to their GP. So*,* basically*,* they’d get referred to the service accepted*,* but they’re not actually seen by anyone. So*,* you’re not going to start them on medication because […] it’s not safe because you’re not reviewing them.*” (S9, Speciality Doctor).

The survey found variation around whether follow-up plans (e.g., discharge from ED services to GP or mental health services) after treatment with olanzapine influenced their prescribing: almost half (7/ 15) agreed, 4 disagreed and 4 remained neutral.

### Theme 3: Limited Service Capacity

Service capacity, being under-staffed and/or having long waiting lists, presented as a major barrier to recruitment as staff reported limited time to discuss study involvement with SUs/families. Almost all indicated their service lacking sufficient resources to fully engage with OPEN. Limited contact with psychiatrists restricted opportunities for participant recruitment and emphasis on short term treatments left some staff feeling uncomfortable with suggesting or prescribing olanzapine.

*“I think the main barrier was when we start olanzapine it’s usually for the most unwell people. And the barriers to the consent*,* the forms*,* everything. You don’t really have the time to work through it because there’s such a sort of bit of a rush to get that person onto the medication to try and avoid an admission and all those other things.”* (S12, Consultant Psychiatrist).

*“CAMHS are really overwhelmed obviously*,* so that’s really*,* really difficult. There’s a long waiting list*,* etc. So*,* that’s always tricky because you need to be mindful. If […]you’re wanting to do only a short piece of work with them*,* you might think*,* “Oh*,* I’m not actually going to prescribe olanzapine because then I won’t be able to discharge them”.* (S1, Speciality Doctor)

Clinicians also noted the cost incurred to sites for study investigations exceeding the compensation received. Services had limited resources (both human/staff and physical/equipment/space/access to investigations), which shaped their practice and hindered their contribution to research.

One interviewee reported *“I’ll invite the researchers to present it to the team. […] I’ll give them regular reminders of engaging with the research. But the reality is they’re really busy clinicians and their minds are on seeing the next patient […] and being realistic*,* I think […] to discuss a research study with the family is probably bottom of their list of things to do.”* (S10, Registered Mental Health Nurse). Quoting this, the survey asked clinicians about service capacity for research. Many (7/15) who were not involved with OPEN disagreed with this quote, whereas most (3/4) clinicians involved in OPEN reported that their team’s workload had increased with the study. This might reflect the difference between anticipated and actual difficulties of such clinical research.

As we identified differences in routine clinical practice around olanzapine for AN, the survey explored some of those practices to inform standards and future study designs. When asked about the standard baseline investigations before initiation of olanzapine, all responders (7/7) chose full blood count, liver function tests, electrolytes; followed by renal function (6/7); lipid profile, bone profile, glucose/haemoglobin A1, creatine kinase and ECG (5/7); prolactin (3/7); and TSH (1/7). None of the responders picked GGT, LH, FSH or pregnancy test. Regarding monitoring frequency for bloods after initiation, responses varied between every week (3/10), every fortnight (2/10), every month (3/10), every three months and every six months (1/10). Monitoring frequency for ECG post-initiation also varied between every week (1/7), every month (5/7) and every six months (1/7).

When asked about workload impact of any potential safety monitoring guidelines or policies for olanzapine in AN, six out of 15 responders of the survey felt that their workload would increase, whilst three felt it would decrease, and two remained neutral. Most doctors (4/7) anticipated no change in their prescribing practice in case of such guidelines, whereas two anticipated some change and one a decrease.

#### Uncertain roles and responsibilities

Many felt that internal processes within teams, and ambiguity regarding roles and responsibilities for OPEN created barriers to recruitment with some staff uncertain about aspects of the study design, including eligibility criteria and screening.

*“[…] Sometimes I wasn’t sure where my role lies. Should I really be encouraging them to start the olanzapine and joining the study? […] But I didn’t feel that comfortable encouraging someone to join a study if they don’t want to. But because I understand how important research is*,* I want to be that person*,* but because I’m not one of the researchers*,* I didn’t know where my role lay within that.”* (S9, Speciality Doctor).

Some clinicians suggested that a prolonged recruitment period may have been helpful, as organising these roles proved time-intensive, especially within limited-service capacity.

*“When I saw the study come through*,* my head went brilliant*,* I am all for that. In reality*,* […] juggling lots of different hats*,* senior hat*,* prescribing hat*,* therapy hat*,* supervisor hat*,* so all these different hats whilst trying to remember about patients that could meet the pathway for olanzapine”* (S11, ED Specialist Nurse).

Clinicians (3/3) involved with OPEN reported in the survey that finding enough time for informed consent has been challenging in their setting, and two out of three did not feel confident enough to complete informed consent with participants despite all three clinicians reporting confidence in explaining patients and families about rationale for olanzapine.

### Theme 4: Study eligibility Criteria

The inclusion criteria for SUs interested in participating in OPEN were also identified as a barrier to recruitment, for example, many SUs appeared to have been prescribed olanzapine already. Other exclusion criteria such as self-harm, suicidality, age or previous weight gain were mentioned as barriers by some.

#### Current use of olanzapine

The severity of illness and urgent need for introducing olanzapine were frequently mentioned as a barrier to recruitment. Some potential participants were on the medication prior to reaching their service and therefore failed eligibility, while others needed olanzapine promptly due to illness severity. Two described this as a change of practice, arising from the pandemic, and a response to increasing numbers of acute presentations requiring urgent intervention.

*“The issue with recruiting is […] that olanzapine is being prescribed because of just everything being in a crisis*,* you know? Probably COVID having to do with it. Because we get YP who more unwell*,* more underweight*,* need kind of earlier intervention. Whereas I think before […]*,* you would be thinking about olanzapine months after they’ve been in.”* (S1, Speciality Doctor).

#### Other aspects for exclusion (self-harm, suicidality, -2 kg weight gain, age)

Some mentioned self-harm or suicidality as common in the SU population, excluding them from OPEN. Others mentioned age-related exclusion. Two clinicians mentioned that initial weight gain often related to re-hydrating SUs upon admission to their service meant that they had to be excluded.

Pregnancy, breastfeeding and unwillingness to commit to contraceptive measures were also exclusion criteria. Furthermore, the Ethics Committee asked for introduction of regular serum pregnancy tests into the study for all participants. Following feedback from recruiting clinicians about perceived difficulties in talking about these with potential participants and families, the survey asked clinicians about serum pregnancy tests and contraceptive measures for research participants on olanzapine for AN. Concerning their practice of performing routine pregnancy tests for sexually *inactive* patients on olanzapine for AN, almost all (15/17) responded “never” and two “ad hoc”. For sexually *active* patients, most (9/17) still responded “never” and eight “ad hoc”. When asked about how comfortable they would feel about requesting regular pregnancy tests for a research participant, five out of 23 responded “uncomfortable”, eight “comfortable” and ten remained neutral. When specified for sexually *inactive* research participants, most of them (12/17) responded “uncomfortable”. Most (3/4) psychiatrists involved with OPEN reported being uncomfortable and one slightly comfortable with requesting regular pregnancy tests: an adult psychiatrist who was uncomfortable noted “*Because it is not common practice*,* and our patients are often quite young and not sexually active*”. This was mirrored by another child and adolescent psychiatry consultant, *“As patients were young and not sexually active”*. Another psychiatrist noted *“I was mostly comfortable about requesting pregnancy tests for the research study because it was part of the ethics and safety monitoring arrangements in the context of clinical research. I don’t think for some of those participants I would have asked for pregnancy tests if I was only involved with them in the context of clinical care (no research)”*.

## Discussion

Despite availability of evidence-based psychological treatments, AN has moderate rates of remission and high risk of mortality [18]. Antipsychotics continue to be routinely prescribed off-label for AN despite lack of a strong evidence base for their efficacy and safety. Yet, the OPEN study struggled with recruitment, adherence and retention [2, 6]. This study allowed an examination of possible reasons for failure from clinicians` perspectives.

Our first theme focused on perceived concerns of SUs/families around olanzapine, mirroring findings from interviews with SUs/families themselves [19]. This included fear of weight gain, stigma around “antipsychotics”, and anxieties arising from the lack of evidence or understanding of how olanzapine effects change. Despite barriers to recruitment, clinicians expressed confidence explaining the rationale for olanzapine in both the survey and interviews, noting the importance of providing clear information around medication with a balanced view on its usefulness and possible side effects, and including family and loved ones in discussion.

The second theme focused on prioritising person-centred care and SUs’ and families’ individual needs and concerns. Almost all underlined the importance of introducing olanzapine *only* if it were appropriate at that stage of SU’s treatment and would not jeopardise therapeutic alliance. This presents obvious challenges for recruiting participants within the narrow confines of an RCT. Concurrently, positive rapport was seen as beneficial not just for engagement in treatment and recovery process, but also enhancing the likelihood of SUs trying a new treatment, including olanzapine, and the study, mirroring existing research [20].

There was a widespread desire among clinicians for a greater evidence base on safety and efficacy of olanzapine for AN with some specific concerns about its use in a younger, developing population. Nevertheless, clinicians were noted to utilise olanzapine with limited current evidence, including anecdotal/individual evidence. It is probable that this was underpinned by a careful assessment of potential risks versus benefits of olanzapine in a young person with AN whose health has already been severely affected by a debilitating and severe illness, with well-known poor long-term outcomes, and having exhausted other options.

Clinicians should offer a balanced view on possible effects of olanzapine on potential therapeutic targets for patients with AN, e.g., anxiety, concentration, sleep, and anorexic thoughts [21]. This resonates with what clinicians had observed with regards to effects of olanzapine as well as findings from the largest RCT to date, i.e., lower rates of difficulties with concentration, agitation and sleep in the olanzapine group compared to the placebo arm [6]. A more nuanced discussion with patients and families around how olanzapine could help, promises to support well informed decisions.

Furthermore, reported reasons for olanzapine initiation reflected previous views in expert reviews that antipsychotics are thought to be useful for AN through multiple potential mechanisms [22]. These include helping to reduce the intensity, rigidity and obsessiveness of ED cognitions, affective and anxiety symptoms, irritability, aggression, and physical hyperactivity. Sleep and weight restoration are achieved probably through a combined effect on the above as well as an increase in appetite and food intake.

The impact of limited service capacity was critical for teams’ ability to offer olanzapine and study participation. Post-pandemic staff shortages meant that services were running at reduced/restricted capacity, resulting in fewer patients being approached/recruited. Also, heterogeneity of ED service setup across the country possibly meant that some clinicians refrained from offering olanzapine, despite clinical appropriateness, due to their inability to regularly review and monitor its use. We noted some differences in routine clinical practice across teams/clinicians around baseline investigations and monitoring of olanzapine for AN and how these could have led to poor recruitment and/or adherence to study procedures. This contradicted our initial expectation that study procedures would not differ significantly from standard practice in most sites and would not cause additional burden/workload for recruiting teams.

Several concerns were raised about eligibility criteria, and these were generally considered as barriers to recruitment. Clinicians identified multiple YP in their services that were already on, or were being started on, olanzapine but would not have been eligible due to rather strict criteria and procedures. These included providing ample time for informed consent, undertaking screening and baseline assessments, self-harm and suicidality being common in AN yet exclusion criteria, the amount and time period for lack of weight gain, having to request regular pregnancy tests in a population not subject to this in routine clinical practice, and more severe initial presentations in current setting generating clinical urgency and motivation to start olanzapine promptly.

These findings shed light on important issues in clinical practice, study design and implementation in this delicate field, particularly when complemented by perspectives of YP and their families [19].

### Strengths and limitations

Involving lived experience throughout this study in the form of PPI and research staff is a major strength that helped reduce power imbalances and providing SU insight. Disclosure of lived experience of nine clinicians completing the survey offers further diversity of perspectives. This is in line with the previously reported high rates (up to 47.5%) of lifetime lived experience among ED clinicians internationally [23]. Bachner-Melman et al. (2021) discuss that the prevalence of clinicians with lived experience may contribute to a lack of g objectivity and risk of being triggered, however, their deep experiential understanding and empathetic, non-judgmental approach were highlighted as strengths.

An online survey, by design, would attract a biased sample, however, we aimed to gather views from clinicians immersed in ED to widen our understanding of clinicians’ perspectives on acceptability and feasibility of a clinical trial of olanzapine for YP with AN, and the survey allowed us to extend our sample to different ED settings, regions and professionals.

Furthermore, we could compare views of clinicians who were involved with OPEN and those who were not. There were some differences between those two groups; for example, considering the impact of limited/reduced capacity of ED services in accommodating clinical research activities. Clinicians who were naive to study procedures appeared less aware of potential increase of workload as a clinician/team. This was also seen/experienced in multiple clinical sites completing their local capacity and capability assessments, yet not being able to recruit any participants or complete study procedures.

Finally, triangulating interview and survey data increased confidence in the results, allowing us to combine the detail of individual experiences in interviews and breadth of perspectives in surveys [24].

## Conclusions

There was consensus among clinicians that more research evidence was needed for olanzapine in YP with AN, including its effect not only on weight gain but also psychopathology, and safety. We anticipate olanzapine will continue to be prescribed clinically as an off-label pharmacological treatment for AN, with patient reluctance remaining an important consideration. Future studies will need to clarify for which patient characteristics olanzapine might be most useful, significantly facilitating decision making of all stakeholders, clinicians, patients and families alike.

This study on clinicians’ perspectives, combined with the perspectives of YP and carers/families [18] on acceptability and feasibility of a clinical trial of olanzapine for AN, works synergistically to help delineate important themes to improve the effectiveness of therapeutic relationship between clinicians and patients/families and building of an evidence base. Creating a clinician’s checklist as a guide for consideration ahead of conversations with SUs around olanzapine might prove useful in clinical encounters to encourage a person-centred approach for prescribing olanzapine and challenge potential power dynamics perceived by SUs.

When trying to produce evidence base on effectiveness of treatments, eating disorders have repeatedly suffered from the requirements of RCTs with inconclusive results that could not further inform clinical practice [22, 25]. An example of a prospective study to test safety during olanzapine treatment has been published by Karwautz et al. (2023); measuring olanzapine serum levels and side effects longitudinally in sixty-five adolescents with AN [26]. Notably, qualitative methodologies and increased inclusion of lived experiences can be used to generate hypotheses about treatment approaches, and adapted observational designs employing large treatment databases of patients in routine clinical care settings to derive safety and effectiveness information for treatments that could not be thoroughly investigated in RCT designs [25]. For informing future clinical research, we recommend facilitating more flexible clinical study designs utilising data from routine clinical practice with olanzapine for AN with consideration of the possibility that an RCT might not be the best fit to study safety and efficacy of olanzapine in YP with AN.

## Electronic supplementary material

Below is the link to the electronic supplementary material.

Supplementary Material 1

## Data Availability

The data that support the findings of this study are available on reasonable request from the corresponding author, VK. The data are not publicly available due to their containing information that could compromise the privacy of research participants and institutions.

## Abbreviations

AN: Anorexia Nervosa
CAMHS: Children and Adolescent Mental Health Services
ED: Eating Disorder(s)
NHS: National Health Service
OPEN: Olanzapine for young PEople with aNorexia nervosa
RCT: Randomised Controlled Trial
SU: Service User
UK: United Kingdom
YP: Young people
