# Supplementary material for: A feasibility trial of olanzapine for young people with Anorexia Nervosa (OPEN): clinicians’ perspectives
Source: J Eat Disord. 2024 Sep 27;12:146. doi: 10.1186/s40337-024-01106-9 (PMC11429100; doi:10.1186/s40337-024-01106-9)
Supplement: Supplementary file 1 — Supplementary Material 1 [file 40337_2024_1106_MOESM1_ESM.docx]

**Topic Guide – Staff Interviews**

Aim of the interviews:

Recruitment/Baseline

- To understand how staff are presenting the study to participants
- To understand how staff view and/or predict participant engagement
- To understand what advantages and disadvantages staff hope for and/or predict for participants
- To outline staff’s understanding of the participants’ decision-making process
- To explore any concerns staff might have related to the study

Follow-up

- To understand how staff experience participant engagement
- To understand differences in treatment development across participants
- To understand what factors staff think influence the study progress

Topic guide

1. = Broad topics | • = Main questions | = follow up questions/prompts
2. Overall experience of staff working in an eating disorders service

- Can you tell us about the setting that you work in and how you support young people with anorexia nervosa?
- What type of treatment do you usually provide as a first-line approach?
- How might treatment develop from there?
- Do you have experience with different therapeutic treatments? (Medication, psychotherapy, family therapy, interventions for carers, dietetics, occupational therapy, nursing interventions, peer support)
- Any other therapies? VR, self-help, apps.
- What aspects of care do you find most important for this population?
- Treatment of specific symptoms or difficulties only (e.g., mood, anxiety, obsessions, compulsions, rigidity, concentration, anorexic voice, appetite increase, weight gain, gastrointestinal symptoms, being secretive) or combinations of interventions?
- Do you think any of these aspects need more intense/extensive support than others?
- What are some challenges you experience in your work with young people with anorexia nervosa?
- Any differences between younger age group and adolescents vs older age groups?

1. Specific experience of staff with olanzapine in supporting young people with anorexia nervosa

- What are your thoughts about using olanzapine with younger people with anorexia nervosa?
- What are your hopes on how it could help?
- Improving symptoms (e.g., mood, anxiety, obsessions, compulsions, rigidity, concentration, anorexic voice, appetite increase, weight gain, gastrointestinal symptoms, secrecy vs. openness)?
- broader psychosocial benefits of olanzapine (e.g., relationships, returning to community/school/work)?
- How do you think olanzapine can help with some of the challenges mentioned above/earlier regarding treating young service users with AN?
- Have you experienced any benefits with this medication and service user group previously?
- Do you have any concerns about using olanzapine with this population (younger age group and adolescents)?
- What do you think of potential side effects of olanzapine? (e.g., sleepiness, weight gain, appetite increase, GI symptoms)
- Other concerns?
- How do you think this might affect the experience of participants?
- How do you think this might affect acceptability and adherence of participants taking olanzapine?
- How do you think this might influence your work and your interactions with service users on this trial?
- How do you think young people themselves might feel about taking olanzapine?
- Do you think this might differ across age groups?
- How do you think families are likely to respond?
- Do you have any experience with previous service-user feedback or self-reported outcomes with olanzapine?
- Have you used any other medication for AN and what has that experience been like?
- How does it compare to olanzapine?
- Do you have any other considerations when thinking about medication for AN, e.g., comorbidities, service user involvement/preference?
- Any other concerns or comments?

1. Staff perceptions around and understanding of the OPEN study

Summary of OPEN: A feasibility study to examine whether it is attainable and beneficial to conduct a trial examining the benefits and harms of prescribing atypical antipsychotics to young people with anorexia nervosa who have not responded to first-line treatments.

Service users aged 12-24 are seen at baseline, at 8 weeks, 16 weeks, 6 months, and 12 months after start of examination. Participants are diagnosed with AN or atypical AN, are receiving olanzapine alongside treatment as usual (inpatients, outpatients, or day programme) in which they struggled to gain 2kg or more within last month of therapeutic engagement.

Qualitative exploration will take place at baseline, after 16 weeks, and 12 months.

- Do you feel well-informed about the study design and purpose?
- What are your initial thoughts on the study design?
- What do you think will work/works well?
- What do you think might/does prove challenging?
- How do you think the trial might affect/has affected your work specifically? (e.g., workload, interpersonal interactions, colleagues’ interventions)
- Positive aspects?
- Challenging aspects?
- How do you feel about recruiting participants to the trial?
- What do you think is an ideal way of approaching potential participants and why?
- What do you think might prove challenging or difficult?
- Have you had any difficulties when recruiting participants so far?
- How do you think service users will feel about participating in the trial?
- How do you think families and carers will feel about their loved one participating in the trial?
- Do you have any questions or concerns about your involvement in the trial?
- How do you think the wider research team could support your service with participating?
